# Accuracy of unguided and ultrasound guided Coracohumeral ligament infiltrations – a feasibility cadaveric case series

**DOI:** 10.1186/s12891-020-3153-4

**Published:** 2020-02-28

**Authors:** John L. Pape, Mathieu Boudier-Revéret, Jean-Michel Brismée, Kerry K. Gilbert, Detlev Grabs, Stéphane Sobczak

**Affiliations:** 1 Department of Physiotherapy, University Hospital of North Tees, Stockton on Tees, UK; 2 Centre hospitalier de l’Université de Montréal, Montréal, QC Canada; 3 Department of Rehabilitation Sciences and Center for Rehabilitation Research, School of Health Professions, Texas Tech University Health Sciences Center, Lubbock, TX USA; 4 Research Unit in Clinical and Functional Anatomy, Départment d’anatomie, Université du Québec à Trois-Rivières, Trois-Rivières, QC Canada

**Keywords:** Glenohumeral, Adhesive capsulitis, Injection, Coracohumeral ligament, Shoulder, Corticosteroid

## Abstract

**Background:**

Coracohumeral ligament (CHL) thickening, contracture, and fibroplasia have been identified in glenohumeral idiopathic adhesive capsulitis (GHIAC). The CHL is the main structure responsible for the range of motion limitations. Favorable outcomes have been reported with CHL surgical release. Intra-articular glenohumeral joint corticosteroid infiltrations are utilized to disrupt the inflammatory process and reduce pain in GHIAC. The aim of this study was to investigate whether the CHL could be accurately targeted with a periligamentous infiltration.

**Methods:**

A convenience sample of 12 unembalmed cadaver shoulders (mean age: 74.5 years, range 66–87 years) without evidence of previous injury or surgery were utilized in this exploratory double factor feasibility cadaveric (unguided and ultrasound (US) guided) case series. Two clinicians trained in musculoskeletal infiltration techniques carried out the infiltrations on each shoulder with colored latex. One clinician infiltrated without guidance, the other with US-guidance. The injecting clinicians were blinded to the others infiltration procedure and the order was randomized. An anatomist blinded to the infiltration order performed a shoulder dissection and recorded the infiltrate location. Percentage calculation for accuracy of infiltration and a chi-square evaluation of the difference between unguided and US-guided infiltrations was applied.

**Results:**

An accuracy of 75% was achieved for unguided infiltration and 80% for US-guided infiltration techniques. Chi-squared indicated there was no significant difference (*p =* 0.82) between the unguided and US-guided techniques.

**Conclusion:**

US-guided and unguided infiltrations achieved good accuracy targeting the CHL, suggesting infiltrations can specifically and accurately target the CHL. In vivo investigation using such infiltration techniques are warranted.

## Background

Glenohumeral idiopathic adhesive capsulitis (GHIAC) is a common source of pain and disability affecting between two and 5 % of the general population [1]. Although GHIAC has frequently been suggested to resolve in 2–3 years, persistent symptoms have been reported on long term follow up with mild pain and loss of function in 35% of subjects at 4.4 years (range 2–20 years) [1] and pain and stiffness in 50% of subjects at 7 years 9 months (range 2 years 2 months – 11 years) [2].

Bunker [3] suggested that fibroplastic changes in the region of the rotator interval are pathognomonic for GHIAC. Thickening, contracture, and fibroplasia of the coracohumeral ligament (CHL) have been identified in GHIAC [4–8]. The loss of external rotation is regarded as a diagnostic finding for GHIAC [3, 9]. Ozaki et al. and Neer et al. have identified the CHL as the main structure responsible for ROM limitations in GHIAC [4, 5]. The CHL is a capsular thickening extending from the lateral base of the coracoid over the rotator interval and superior aspect of the shoulder, blending with the capsule and inserting into the greater and lesser tuberosities [10, 11]. The CHL is taut in External rotation [10]. Favorable outcomes have been reported in studies targeting the CHL with a variety of interventions including surgical release [4, 5, 12], microadhesiolysis [13] and stretching [14]. The CHL has also been identified as a target structure for manual therapy in GHIAC [15].

Intra-articular glenohumeral joint infiltrations with corticosteroids are frequently used in the treatment of GHIAC for pain reduction and to disrupt the inflammatory process [10, 16–20]. They have been found to offer short-term improvements, with a rapid decrease in pain and increase in ROM in the first 6 weeks after treatment [21]. However, there is no consensus on the site of infiltration [22]. Studies comparing glenohumeral with subacromial corticosteroid infiltrations and oral corticosteroids have similar outcomes [21]. The absence of a superior beneficial effect of corticosteroid delivery within the glenohumeral joint may be attributed to an inability to accurately localize the site of pathology. This has been suggested to be the primary reason for the absence of effective and predictable treatment outcomes in GHIAC [14]. It would seem unlikely that corticosteroid delivered intra-articularly would have an optimal effect on extra-articular CHL inflammation and fibroplastic processes.

To date, no corticosteroid infiltration study has specifically targeted the CHL for GHIAC. As the CHL is often surgically released in cases of GHIAC by orthopaedic surgeons, it would seem imperative to target the ligament using less costly conservative means that could be easily performed by general healthcare providers.

Therefore, the purposes of this study were to investigate (1) whether the CHL could be accurately targeted with a periligamentous infiltration by clinicians trained in musculoskeletal infiltration techniques; and (2) whether there was added value of ultrasound (US)-guided infiltration. The long-term goal of this line of research is to investigate cost effectiveness of this infiltration approach performed by clinicians in patients with GHIAC. The data and methods of this study have briefly been presented at the Physiotherapy UK Conference 2018 [23], this article is to present the novel injection techniques and findings from this study in further detail.

### Design

Exploratory double factor cadaveric (unguided and US guided infiltration) case series.

## Methods

### Subjects

Six unembalmed cadavers (12 shoulders) from the Willed body program at the Départment d’anatomie, Université du Québec à Trois-Rivières were utilized for this study. Exclusion criteria were evidence of arthroplasty, implants, surgery, capsulo-ligamentous or bony injury to the shoulder. The cadavers were frozen at − 18°c and were thawed for 48 h at room temperature (16°c), before the infiltrations were performed. The average age, weight and height of the cadavers were 74.5 years (range, 66–87 years), 57.2 kg (range 40–77.5 kg) and 165.5 cm (range,157-173 cm), respectively.

### Apparatus

Blue and red laboratory grade latex medium (Carolina Biological Supply Company, Burlington, NC, USA) were injected with a 1 ml syringe and 21-gauge needle, 5% acetic acid was injected with a 20 ml syringe and 25-gauge needle. An US (LogiqE, General Electric, Mississauga, ON, Canada) was used for imaging during the US guided infiltrations.

### Experimental procedure

The experimental protocol received approval by the subcommittee ethic board from the Départment d’anatomie, Université du Québec à Trois-Rivières (SCELERA-17-01) and carried out in the Gross Anatomy and the Clinical Anatomy Research Laboratories of Départment d’anatomie, Université du Québec à Trois-Rivières in June 2017. Two primary health care providers trained in musculoskeletal infiltration techniques participated in the study. An extended scope physiotherapist (ESP) specialized in musculoskeletal assessment, which included assessment and treatment using manual therapy techniques, exercise and infiltration techniques for 10 years performed the unguided infiltrations. A recently graduated physical medicine and rehabilitation specialist exposed to US guided infiltrations during his 5 years of residency in physical medicine and rehabilitation performed the US guided infiltrations. Both clinicians injected each shoulder once. The injecting clinicians were blinded to each other’s infiltration technique. The order of infiltration (i.e. US-guided and unguided) was randomized for each shoulder. Between infiltrations, the skin surface was cleaned with a tissue to remove traces of the latex dye so the previous injection site was not visible to the second injector. The color of latex dye used by each injecting clinician was also randomized to blind the dissecting investigator to the injecting clinician. A 0.05–0.1 ml bolus of latex dye was injected. In order to solidify the latex dye, the area was infused with 7-10 ml of 5% acetic acid. An anterior shoulder dissection was carried out by the third investigator, who is an anatomist with 15 years of experience, to determine the location of the dye boluses. The third investigator was blinded to the color of dye used by the two injecting clinicians. Direct contact of the latex dye bolus with the CHL was deemed an accurate periligamentous infiltration. The latex dye was viscous, adhered to the tissues and solidified rapidly on contact with acetic acid. Due to these properties and the small volume delivered in the injection bolus, it was deemed unlikely that the latex dye would be displaced by the infused acetic acid or the subsequent dissection.

### Unguided infiltration technique

**Fig. 1:**
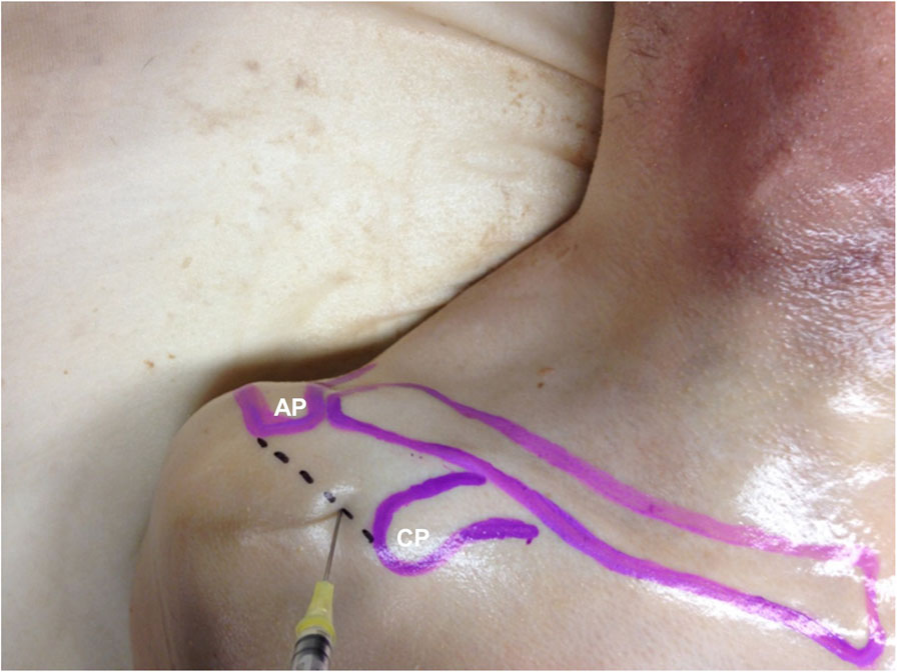
Bony landmarks for unguided Coracohumeral ligament periligamentous injection needle placement of a right shoulder. CP: Coracoid Process; AP: Acromion Process. The needle is inserted 1cm from the lateral tip of the Coracoid Process along a line connecting the lateral tip of the Coracoid Process with the lateral tip of the ventral border of the Acromion Process: dotted line

Anatomical landmarks for the unguided infiltration targeting the CHL were determined using US-guided infiltration and subsequent dissection in a previous pilot study. The lateral tip of the coracoid process, superior aspect of the lesser tuberosity and the lateral tip of the ventral border of the acromion process were located by palpation. The infiltration site was located 1 cm lateral to the lateral tip of the coracoid process along a line connecting it to the lateral tip of the ventral border of the acromion process (Fig. 1). The hypodermic needle was inserted perpendicularly through the skin at this point. The needle was advanced in a posterior direction until the resistance (increased stiffness) to the needle detected the CHL and the bolus of latex dye was delivered.

### US-guided infiltration technique

**Fig. 2:**
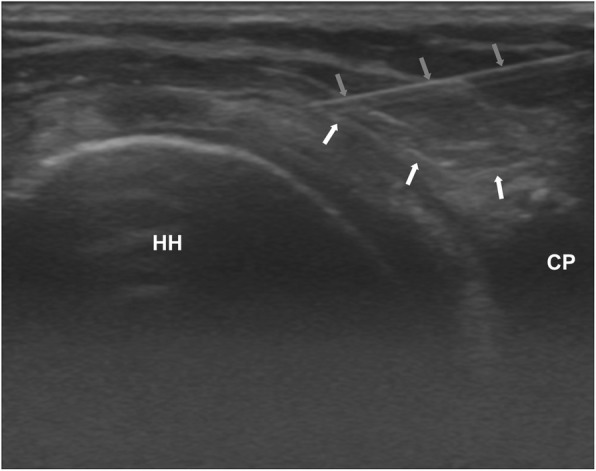
Ultrasound scan guided Coracohumeral ligament periligamentous injection of a right shoulder showing coracohumeral ligament: white arrow heads; HH: Humeral head; CP: Coracoid process; injecting needle: blue arrow heads. Transducer placement over the anterior superior aspect of the shoulder, with the coracohumeral ligament in the long axis

Using the US probe oriented in the sagittal plane of the body, the long head of the biceps was identified in the rotator cuff interval and the humeral head cartilage deep in the long axis. The infiltration was in plane with the US-probe. As the injecting clinician held the US-probe with his right hand and infiltrated with his left hand on the right shoulder, the infiltration was performed in a caudal direction and when it was on the left shoulder in a cephalad direction. The needle was inserted just superficial to the CHL (Fig. 2).

### Statistical analysis

A dichotomous decision of accurate-inaccurate injection was made. The accuracy calculated as percentages by dividing the number of accurate infiltrations by the total number of injections multiplied by 100 for both the unguided and US guided infiltrations. The numbers of accurate and inaccurate injections for both the unguided and US guided infiltrations were assessed for statistical significance using Chi-Square analysis.

## Results

**Fig. 3:**
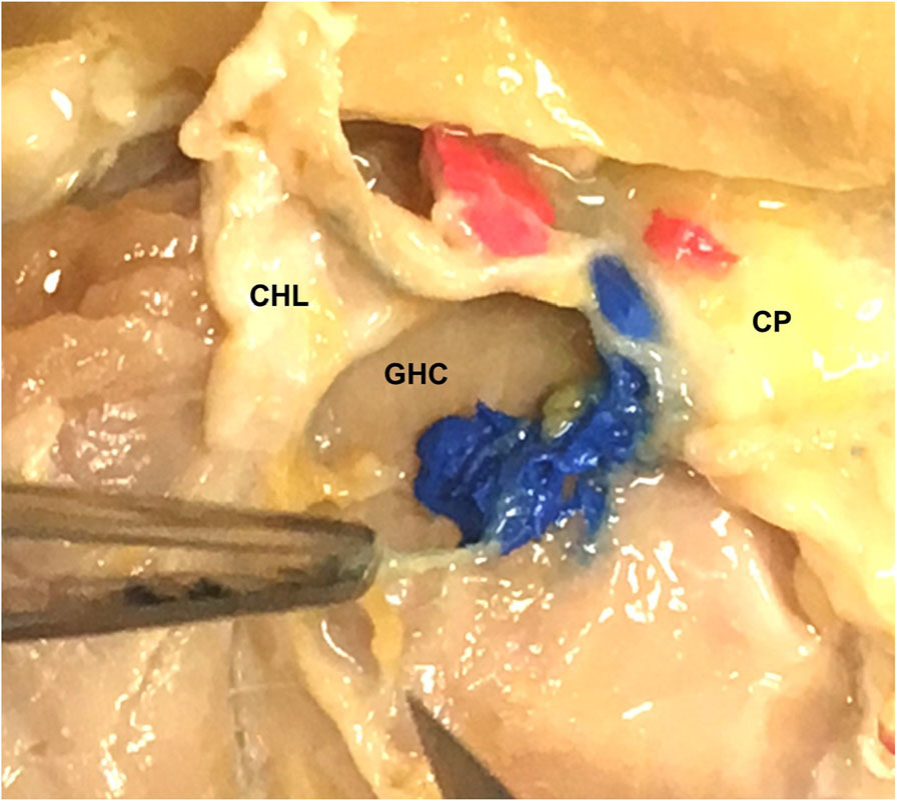
Showing latex dye location following US-guided and unguided infiltrations for a right shoulder, with the blue latex dye following unguided injection in contact and deep to the Coracohumeral ligament and red dye following US-guided infiltration in contact with the anterior surface of the Coracohumeral ligament. CHL: Coracohumeral Ligament; CP: Coracoid Process; GHC: Glenohumeral joint Capsule

The bolus of latex dye was in contact with the CHL in 9 of the 12 unguided infiltrations and 8 of the 12 guided injections (Fig. 3). The accuracy of the unguided infiltrations was 75% and US-guided infiltrations was 67%. A Chi-square test of independence was conducted to assess whether infiltration would be more accurate with unguided or US-guided infiltrations. Yates correction was deemed necessary due to the small number of subjects and more than 20% of the expected frequency cells had counts of 5 or less. For α = .05 the accuracies of unguided and US-guided injections were not significantly different. *Χ*^2^ (Yates correction) (1, *Ν*=24) = 0.00, *p* = 1.00.

Whereas the ESP had previously participated in a pilot study exploring the technique for infiltrating the CHL, the physiatrist had not participated in the pilot study and although experienced in US-guided injections, was unfamiliar with this particular injection technique. The first 2 US-guided infiltrations were not accurate, this was attributed to lack of experience with this injection technique and some initial technical difficulties with an unfamiliar US machine. Therefore, the results from first 2 US guided infiltrations were discarded. Of the remaining 10 US-guided infiltrations 8 were in contact with the CHL, giving an accuracy of 80%. Again, a Chi-square test of independence with Yates correction showed the accuracies of unguided and US-guided infiltrations were not significantly different. *Χ*^2^ (Yates correction) (1, *Ν*=22) = 0.054, *p* = 0.82 (Table 1).

**Table 1:** Descriptive data: accurate, not accurate, total and percentage accurate for US-guided and unguided Coracohumeral periligamentous infiltrations

	Infiltration Type	
	US-guided	Unguided
Accurate	8	9
not accurate	2	3
Totals	10	12
Percentage Accurate	80%	75%

For the 9 accurate unguided infiltrations, in 7 the bolus was in contact with the CHL anterior surface and 2 were in contact with the posterior surface. In all 8 of the accurate US-guided infiltrations, the bolus was in contact with the anterior surface of the CHL.

In the unguided infiltrations that were not accurate, the bolus was located intra-articular in the glenohumeral joint in 2 specimens and posterior to the subscapularis muscle in the third attempt. For the inaccurate US-guided infiltrations the bolus of latex was located intra-articular in the glenohumeral joint.

## Discussion

This is the first study to investigate the accuracy and feasibility of US-guided and unguided periligamentous CHL infiltrations. With an overall accuracy of 75% for unguided infiltrations and 80% for US guided infiltrations would suggest that the CHL could be targeted successfully with this technique in subsequent trials in live subjects with GHIAC. If the CHL can be successfully targeted using feedback (resistance) from the needle in cadavers without obvious shoulder pathology, then the grossly thickened rubbery scar tissue of the CHL in GHIAC [6] should provide increased feedback and assist in accurate placement of the infiltration bolus in these subjects. For intra-articular glenohumeral joint injections the accuracy determined by radiogram of US-guided injections has been reported at 90% and unguided injection at 76.19% [24]. In this study targeting the CHL, US-guided injections were slightly less accurate at 80% while the unguided injections achieved similar accuracy at 75%. The targeting of a thin membranous like structure like the CHL could be regarded as technically more demanding than a relatively large joint space. Despite the expected difficulty with this injection it has demonstrated good accuracy for both US guided and unguided injections.

The good accuracy achieved with the unguided infiltrations, suggests that a pragmatic approach to these infiltrations can be taken. This intervention could be taken by primary care providers without recourse to onward referral to a consultant or for infiltration under guidance. This should offer a timely intervention with reduced delay and costs. Note that the inaccuracy of the first 2 US-guided infiltrations was attributed to technical difficulties with an unfamiliar US device and lack of familiarity with this particular infiltration technique. In the subsequent US-guided infiltrations, the physiatrist then reached 80% accuracy, indicating that US-guided learning occurs quickly for this technique, but necessitates practice. Pre-trial training would have prevented these 2 injections being discarded. A cross over study could have been utilized to examine for the effect of levels of experience on injections accuracy.

For the accurate infiltrations, the majority of the injected boluses were in contact with the anterior surface of the ligament, and two were in contact with its posterior aspect. It is difficult to predict if there is any difference in clinical efficacy with the anterior or posterior location of the bolus. As the rotator interval is also involved in GHIAC [3, 7], it may be expected that delivery deep to the CHL is more effective. For the US guided infiltrations deemed inaccurate and not in contact with the CHL the bolus of latex dye was located intra-articular within the glenohumeral joint. Three unguided infiltrations were deemed inaccurate. In two of these, the latex dye bolus was located within the glenohumeral joint. In the third, the bolus was delivered deep to the subscapularis muscle. Those infiltrations delivered intra-articular within the glenohumeral joint would normally be considered effective for GHIAC. Infiltrations delivered between the glenohumeral joint capsule and the CHL would lie in the rotator interval, a structure implicated in GHIAC. These infiltrations would be considered clinically effective, especially in the light of recent work on collagenase infiltrations with positive outcome attributed to the effect on the rotator interval and the CHL [25].

It has been suggested that infiltration of ligaments is inappropriate as it may lead to rupture [26]. However, the evidence against corticosteroid targeting ligaments is largely anecdotal [27]. The CHL is usually a thin fold of the glenohumeral capsule. It is lined by synovium on its anterior surface and has little resemblance to a true ligament [28]. Concerns about CHL rupture are further allayed by the fact that it is frequently targeted for release by orthopaedic surgeons [4, 5, 12] and torn by manipulation under anaesthesia [6]. Additionally, there may be concerns that the long head of biceps (LHB) tendon may be compromised by an inaccurate infiltration targeting the CHL. During dissection, the colored dye needle tracks through the tissue were clearly visible in many of the injected shoulders. These needle tracks were not in proximity to the LHB. The upper, middle and lower trunks of the brachial plexus, cephalic vein, axillary artery and vein are all located medially and inferiorly to the coracoid process [29, 30] and therefore remote from the infiltration. A similar infiltration approach has been utilized to target the glenohumeral anterior capsule with collagenase infiltration without serious complications [25]. As intraarticular corticosteroid infiltration is advocated in the treatment of GHIAC and may be chondroprotective [31–33], this risk is not incurred. Essentially infiltration targeting the CHL with corticosteroid by an anterior approach may be regarded as a safe infiltration.

Inaccurate corticosteroid placement can compromise therapeutic outcomes of infiltration therapy [34, 35]. It was therefore imperative to demonstrate that the CHL could be accurately and consistently targeted for future studies and therapeutic treatments. The good accuracy obtained with the unguided infiltrations suggests that this technique can easily be carried out in primary care. Although fluoroscopic and US-guided infiltrations have become the gold standard for accuracy, guided infiltrations are frequently unavailable in primary care and onward referral might result in delay and increased expense. However, it should definitely be considered after poor outcomes with previous unguided infiltrations [34, 35]. As the GHIAC response to corticosteroid infiltrations is better in the early stages [18], early infiltration in the primary care setting could shorten the duration of the symptoms and disability with a significant value in terms of reduced morbidity and costs to both the individual and the community [36].

Limitations of the study are the limited numbers of cadaver subjects and also the limited number of injecting clinicians that participated. However it is not uncommon for cadaveric injection studies to be based on a limited number of subjects and a limited number of injecting clinicians [37–39]. A further limitation is the varied levels of experience of the injecting clinicians and specifically varied level of experience with this technique, resulting in the first 2 US-guided injections been discarded. This could have been avoided with pre-trial training. A cross over study could have been utilized to examine for the effect of levels of experience on the accuracy of injections.

Future studies are needed to progress to in vivo infiltrations in subjects with GHIAC including reliability studies with these infiltrations being carried out by other clinicians and studies of efficacy with randomized controlled trials.

## Conclusion

This feasibility cadaveric case series demonstrated that both US guided and unguided periligamentous injections targeted the CHL with good accuracy. This may represent a more specific option for GHIAC treatment than intra-articular infiltrations. Further studies are needed to progress to in vivo infiltrations in subjects with GHIAC and to investigate the reliability of these infiltrations when carried out by other clinicians.

## Data Availability

All data generated or analysed during this study are included in this published article.

## Abbreviations

CHL: Coracohumeral ligament
ESP: Extended scope physiotherapist
GHIAC: Glenohumeral idiopathic adhesive capsulitis
LHB: Long head of biceps
US: Ultrasound
